# Rapid vessel segmentation and reconstruction of head and neck angiograms from MR vessel wall images

**DOI:** 10.1038/s41746-025-01866-x

**Published:** 2025-07-28

**Authors:** Jin Zhang, Wen Wang, Jinhua Dong, Xiong Yang, Shuwei Bai, Jiaqi Tian, Bo Li, Xiao Li, Jianjian Zhang, Hangyu Wu, Xiaoxi Zeng, Yongqiang Ye, Shenghao Ding, Jieqing Wan, Ke Wu, Yufei Mao, Cheng Li, Na Zhang, Jianrong Xu, Yongming Dai, Feng Shi, Beibei Sun, Yan Zhou, Huilin Zhao

**Affiliations:** 1 Department of Radiology, Ren Ji Hospital, Shanghai Jiao Tong University School of Medicine, Shanghai, China; 2 College of Health Science and Technology, Shanghai Jiao Tong University School of Medicine, Shanghai, China; 3 United Imaging Healthcare CO., Ltd, Shanghai, China; 4 Shanghai Clinical Research and Trial Center, Shanghai, China; 5 ShanghaiTech University, Shanghai, China; 6 Department of Radiology, Ningbo Hangzhou Bay Hospital, Ningbo, China; 7 South TaiHu Hospital Affiliated to Huzhou College, Huzhou, China; 8 Department of Neurosurgery, Ren Ji Hospital, Shanghai Jiao Tong University School of Medicine, Shanghai, China; 9 Paul C. Lauterbur Research Center for Biomedical Imaging, Shenzhen Institutes of Advanced Technology, Chinese Academy of Sciences, Shenzhen, China; 10 School of Biomedical Engineering & State Key Laboratory of Advanced Medical Materials and Devices, ShanghaiTech University, Shanghai, China; 11 Department of Research and Development, United Imaging Intelligence, Shanghai, China

**Keywords:** Carotid artery disease, Magnetic resonance imaging, Stroke

## Abstract

Three-dimensional magnetic resonance vessel wall imaging (3D MR-VWI) is critical for characterizing cerebrovascular pathologies, yet its clinical adoption is hindered by labor-intensive postprocessing. We developed VWI Assistant, a multi-sequence integrated deep learning platform trained on multicenter data (study cohorts 1981 patients and imaging datasets) to automate artery segmentation and reconstruction. The framework demonstrated robust performance across diverse patient populations, imaging protocols, and scanner manufacturers, achieving 92.9% qualified rate comparable to expert manual delineation. VWI Assistant reduced processing time by over 90% (10–12 min per case) compared to manual methods (*p* < 0.001) and improved inter-/intra-reader agreement. Real-world deployment (*n* = 1099 patients) demonstrated rapid clinical adoption, with utilization rates increasing from 10.8% to 100.0% within 12 months. By streamlining 3D MR-VWI workflows, VWI Assistant addresses scalability challenges in vascular imaging, offering a practical tool for routine use and large-scale research, significantly improving workflow efficiency while reducing labor and time costs.

## Introduction

Stroke ranks as the second leading cause of death worldwide and remains a primary driver of severe disability and mortality associated with cerebrovascular disease^1,2^. In recent years, 3D MR vessel wall imaging (3D MR-VWI) has emerged as a transformative non-invasive technique for accurately assessing and deep analyzing the cause of stroke^3–6^. Through multi-sequence imaging, 3D MR-VWI can provide rich imaging information, which shines in identifying the etiology^7,8^, determining and tracing vulnerable plaques^9–11^, finding unstable aneurysms^12,13^, and analyzing occlusion characteristics to guide interventional procedures^14,15^.

Image reformation and reconstruction are important in the visualization of arteries vasculature. In the clinical diagnosis, 3D MR-VWI reconstruction remains heavily reliant on manual post-processing so far. Neuroradiologists typically perform curved planar reconstructions (CPR) and multiplanar reconstructions to enhance the visualization of the complex cranio-cervical vasculature^11^. However, the process is highly time-intensive, often requiring over 10 min per patient to reconstruct the entire neck and head arterial pathways. The rising demand for 3D MR-VWI examinations has further exacerbated this burden, straining clinical workflows and introducing potential delays in diagnosis.

Advances in deep learning (DL) offer a promising solution to the challenges of manual image reconstruction. Widely adopted in medical imaging, DL enables fast and precise image segmentation and reconstruction^16–19^. An automated DL-based reconstruction system has the potential to not only alleviate the labor demands of manual processing but also enhance diagnostic consistency and accelerate clinical workflows^20–22^. Several previous studies had made some attempts to use DL for 3D MR-VWI reconstruction^23–26^, while their efforts were limited by small sample sizes, reliance on single manufacture, and narrow population. In this present study, we sought to develop an automatic imaging reconstruction system of 3D MR-VWI based on DL, named VWI Assitant, that can be used with diverse imaging protocols, equipment, populations, including those with vascular occlusion.

## Results

### Patients and image characteristics

**Fig. 1:**
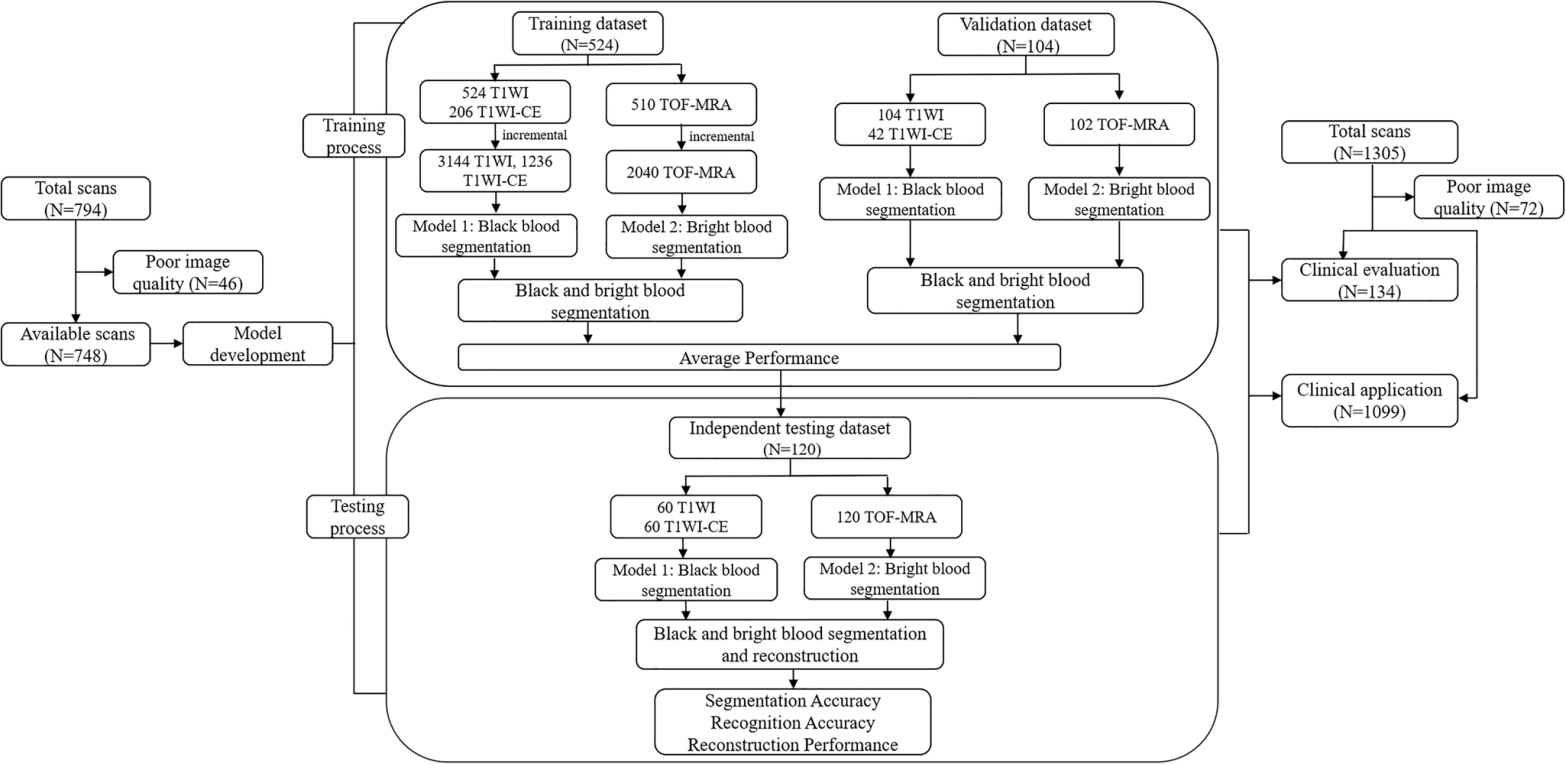
Study design. Data flow chart.

A total of 1981 patients undergoing 3D MR-VWI at four institutions were included in this study. The mean age of the participants was 61 ± 12 years, with 686 (34.6%) being female. The characteristics of patient cohort and the 3D MR-VWI scans used for training, validation, testing, clinical evaluation, and application datasets are summarized in Table 1. The overall experimental design of the workflow diagram is shown in Fig. 1.

**Table 1:** Basic characteristics of enrolled subjects, number of MR images from different manufacturers, and number of different diseases

Parameters	Patient (images) metric
**Patient characteristics**	
Number of patients in training / validating / testing / clinical evaluation and application data sets	524/104/120/134/1099
Male to female	350:174 / 72:32 / 78:42 / 88:46 / 707:392
Age (y)	55 ± 14 / 50 ± 17 / 62 ± 12 / 58 ± 13 / 62 ± 11
Different manufactures	Number of patients in training / validating / testing / clinical evaluation and application data sets
Philips	136 / 21 / 23 / 54 / 824
Siemens	103 / 20 / 25 / 36 / 178
United Imaging	260 / 56 / 65 / 30 / 14
GE	25 / 7 / 7 / 14 / 83
Different diseases	Number of patients in training / validating / testing / clinical evaluation and application data sets
Normal or Atherosclerotic Stenosis (0-99%)	200 / 36 / 42 / 63 / 685
Occlusion (100%)	220 / 40 / 45 / 41 / 238
Others (aneurysm, dissection, vasculitis and so on)	104 / 28 / 33 / 30 / 176

### Algorithm performance

**Fig. 2:**
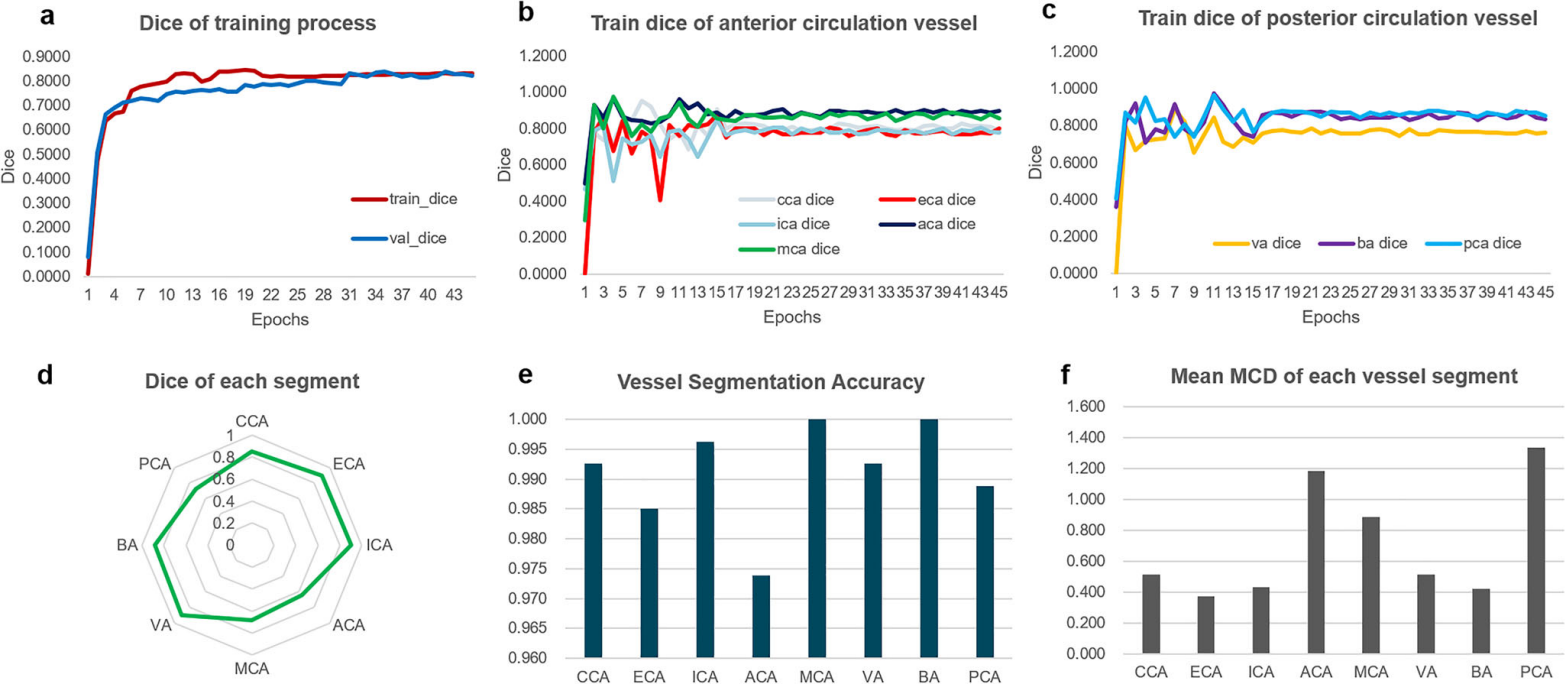
Performance of vessel segmentation and recognition for 3D MR-VWI during training and testing phases. **a** Dice coefficient variation throughout the training and validation process. **b** and **c** Changes in Dice coefficient for anterior and posterior circulation vessels during the training process. **d** Segmentation test results for different vessel segments. **e** Segmentation recognition accuracy for various vessel segments. **f** Test results for centerline extraction of different vessels.

During the model training phase for vascular segmentation and recognition, the training curves for the anterior and posterior circulating arterial vessels converged after around 2500 epochs. The study reported the Dice coefficient for the coarse segmentation and the vascular recognition accuracy of each vascular segment, as shown in Fig. 2. The average centerline distance (in mm) between the automatically extracted vascular centerline by the algorithm and the doctor-annotated centerline was as follows: 0.5149 (CCA), 0.3725 (ECA), 0.4289 (ICA), 1.1839 (ACA), 0.8843 (MCA), 0.5123 (VA), 0.4224 (BA), and 1.3334 (PCA), as shown in Fig. 2.

The study also presented the loss function variation curve during the model training and validation, demonstrating the accuracy of coarse segmentation and vessel recognition during the testing phase, as well as the final test results for the extracted blood vessel centerline. Furthermore, the study evaluated the impact of different MRI sequences combinations on model performance using data from 83 randomly selected subjects. The combination of T1WI, TOF-MRA, and T1WI-CE sequences performed the best, as shown in Table 2.

**Table 2:** Performance of different combinations of MR sequences (*n* = 83)

Combinations			Segmentation	Centerline		Reconstruction	
T1WI	TOF	T1WI-CE	Dice	Mean HD (mm)	MCD (mm)	Qualified rate	Time cost (s)
√			0.820 (0.811, 0.828)	6.61 (6.17, 7.05)	1.38 (1.25, 1.51)	77.1%	20.9
	√		0.650 (0.620, 0.681)	12.21 (10.65, 13.78)	2.69 (2.32, 3.05)	63.9%	17.6
√	√		0.827 (0.819, 0.835)	5.89 (5.45, 6.32)	1.24 (1.11, 1.37)	84.3%	38.1
√		√	0.823 (0.815, 0.831)	6.53 (6.08, 6.98)	1.35 (1.22, 1.48)	86.7%	25.3
√	√	√	0.829 (0.822, 0.836)	5.74 (5.33, 6.14)	1.21 (1.10, 1.32)	92.8%	53.7

Numbers in parentheses are the 95% CIs.

### Clinical evaluation of VWI Assistant

**Fig. 3:**
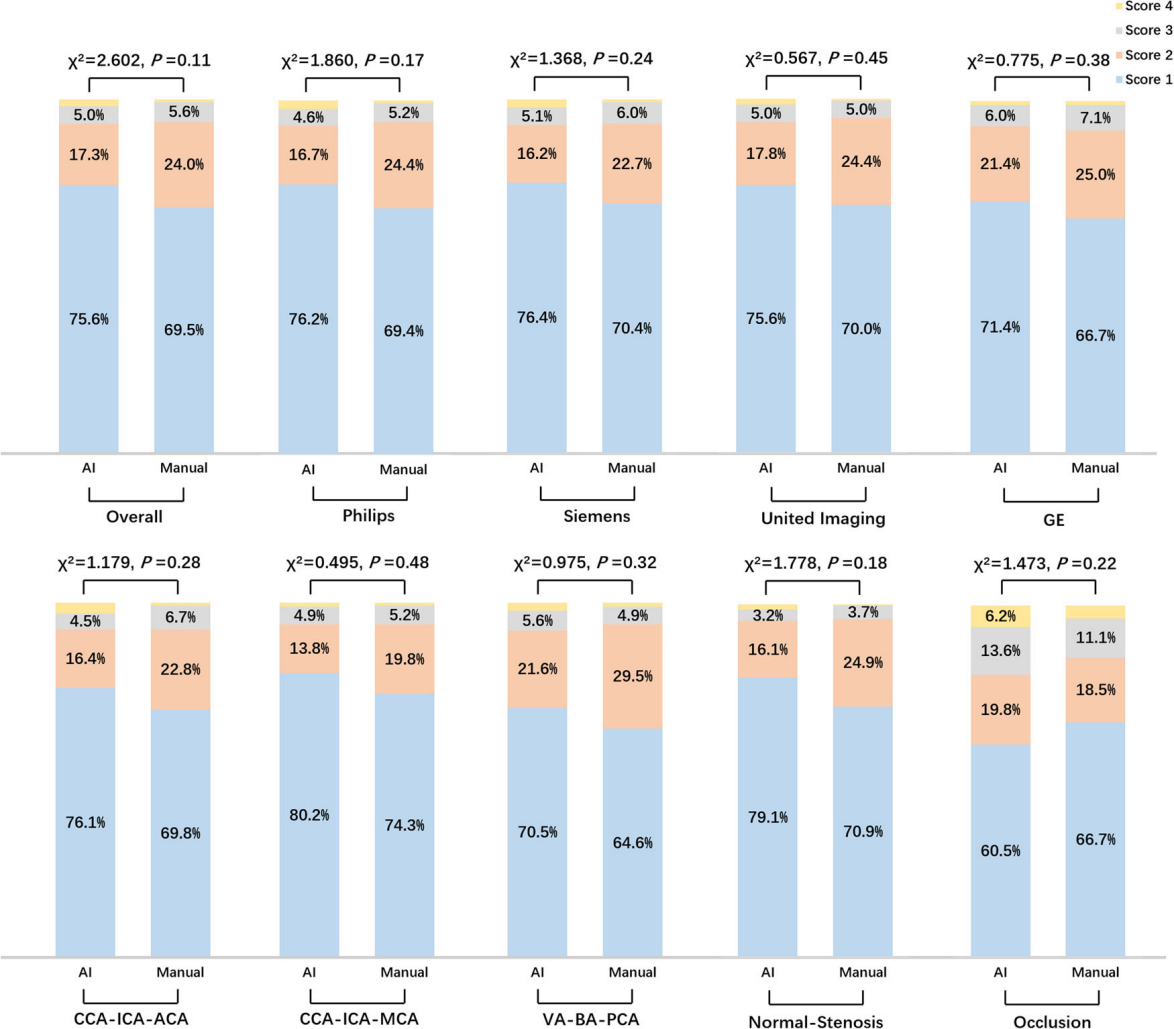
Comparison of 3D MR-VWI reconstruction quality between VWI Assistant and manual postprocessing. Distribution of 4-score ratings across the overall clinical evaluation dataset, as well as for different manufacturers, target vessels, and disease types.

**Fig. 4:**
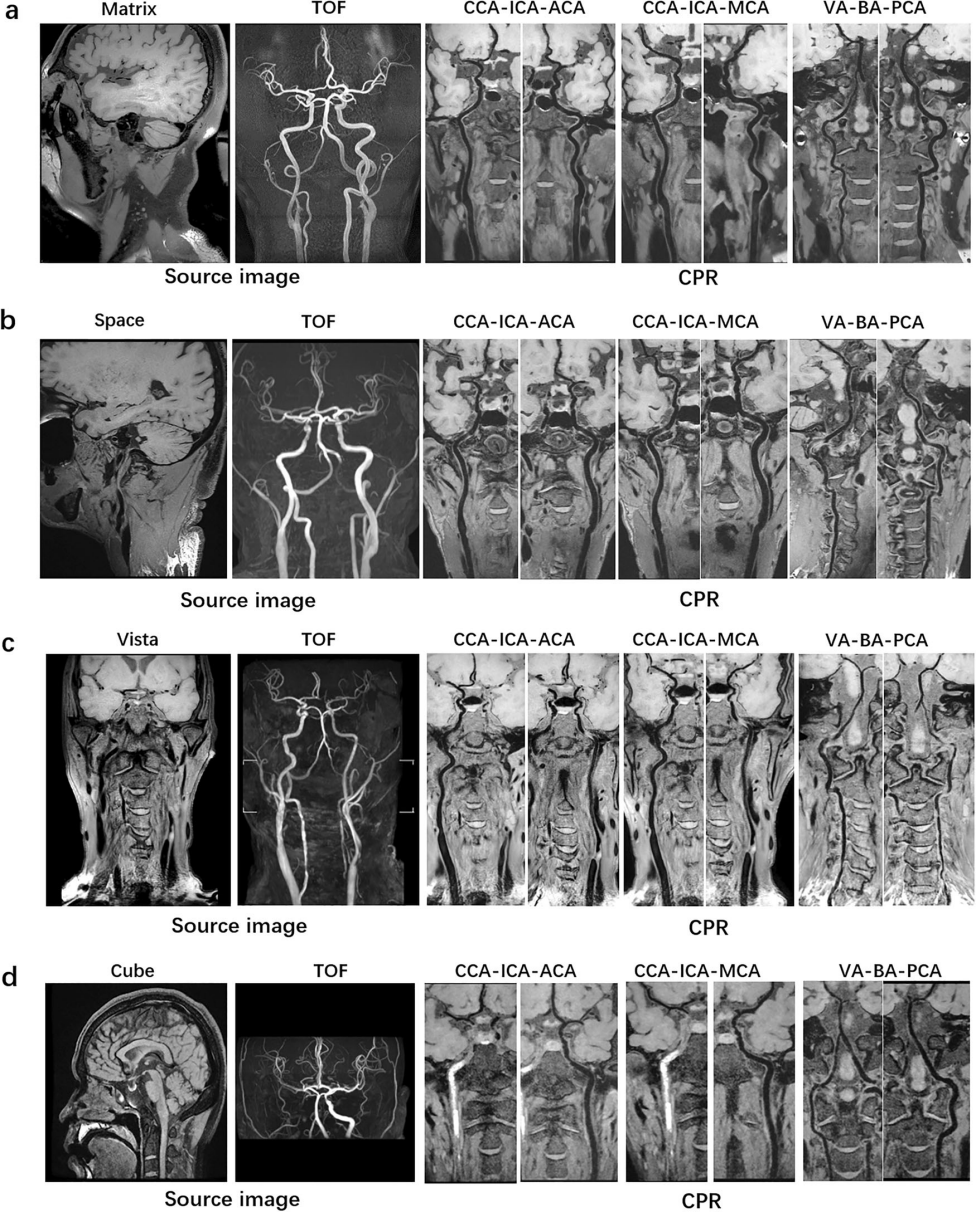
Presentation of reconstructed images generated by VWI Assistant from various black-blood sequences across different manufacturers. **a** Matrix of United Imaging. **b** Space of Siemens. **c** Vista of Philips. **d** Cube of GE.

**Fig. 5:**
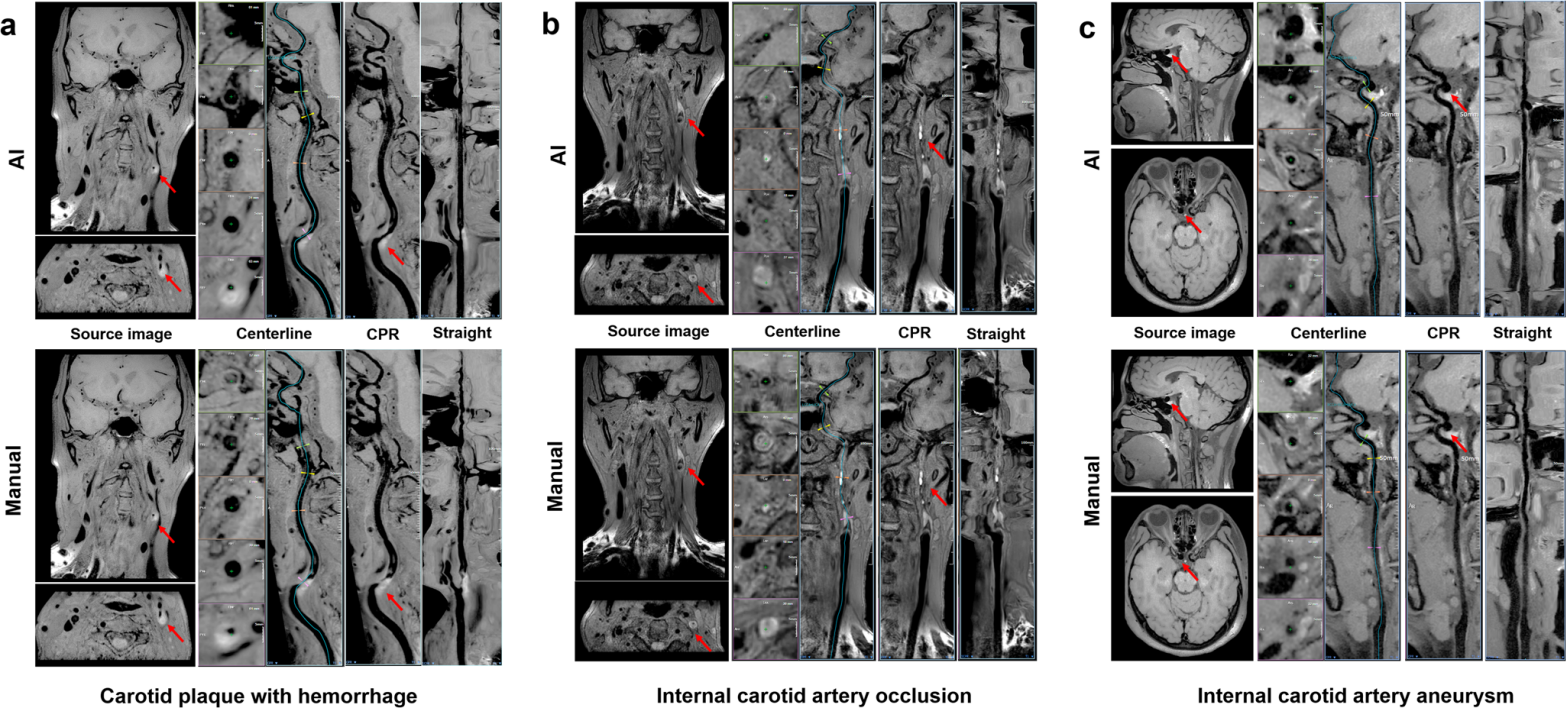
Reconstruction images of carotid arteries with different diseases processed by both VWI Assistant and manual postprocessing. **a** Atherosclerotic plaque with IPH at the bifurcation of the left carotid artery. **b** Occlusion of the left internal carotid artery from the C1 to C2 segment. **c** An aneurysm in the C7 segment of the right internal carotid artery. Both VWI Assistant and manual outputs demonstrated excellent quality, with AI-extracted centerlines showing smoother contours compared to manual extraction.

**Fig. 6:**
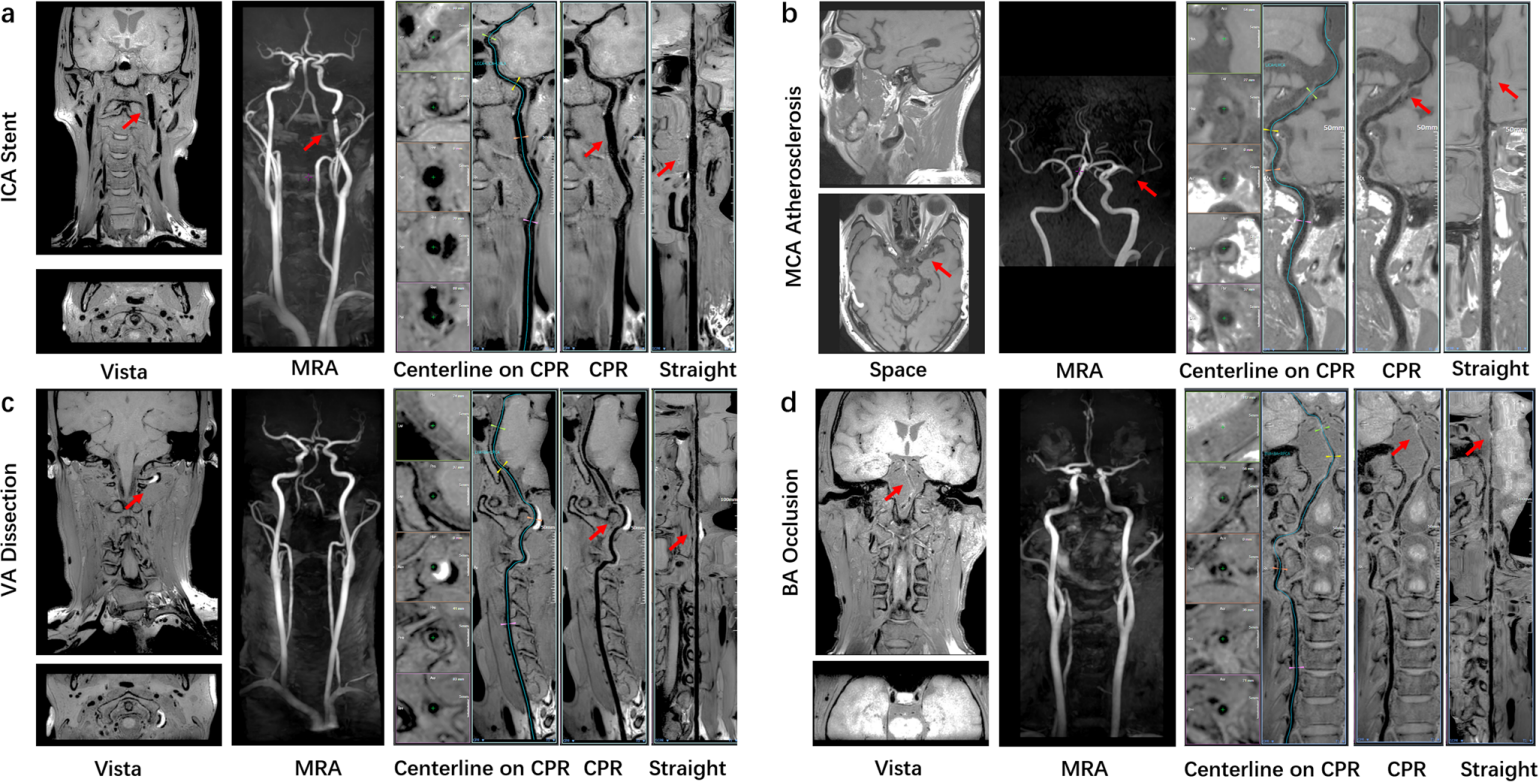
Presentation of reconstruction images of carotid artery with stent and other target arteries by VWI Assistant. **a** A stent placed in the left internal carotid artery. **b** Severe stenosis in the left middle cerebral artery. **c** Intramural hematoma in the left vertebral artery. **d** Occlusion in the basilar artery.

We assessed VWI Assistant for its ability to automatically reconstruct arteries from raw 3D MR-VWI data, assessing whether the reconstructed image quality met the diagnostic needs and comparing its performance to manual reconstruction. The overall qualification rates for the deep learning model and manual output were 92.9% and 93.5%, respectively. When evaluating the qualification rate across MR manufacturers, artery segments and diseases, no statistically significant differences were found between VWI Assistant and manual reconstruction (all *p* > 0.05), as shown in Fig. 3. Figure 4 presents reconstructed images generated by VWI Assistant from various black-blood sequences across different manufacturers. Figure 5 shows the high-quality reconstructions of different carotid artery diseases of intraplaque hemorrhage (Fig. 5a), occlusion (Fig. 5b), and an aneurysm (Fig. 5c), both from VWI Assistant and manual reconstruction. Figure 6 presents additional cases of cranio-cervical diseases reconstructed by the VWI Assistant during clinical evaluation, including a carotid stent (Fig. 6a), atherosclerotic plaque on the middle cerebral artery (Fig. 6b), vertebral artery dissection (Fig. 6c), and basilar artery occlusion (Fig. 6d). These cases were successfully reconstructed, enabling clear visualization of the lesions.

Among the 804 arteries from 134 patients, 57 (7.1%) arteries were scored as unqualified by VWI Assistant reconstruction. Of these, 42 cases (40 scored 3, 2 scored 4) had poor-quality reconstruction due to the centerline deviating by more than 25% from the target vessel. Incorrect vessel segmentation and recognition were observed in 11 cases, including 4 instances where veins were misidentified as arteries. Vessel unrecognition occurred in 4 cases. In contrast, 52 arteries (6.5%) were scored as unqualified by manual reconstruction (45 scored 3, 7 scored 4). Of these, 49 cases (45 scored 3, 4 scored 4) exhibited poor-quality reconstructions with a centerline deviating by more than 25%. Additionally, 3 cases were deemed unqualified due to vessel tracing issues.

**Fig. 7:**
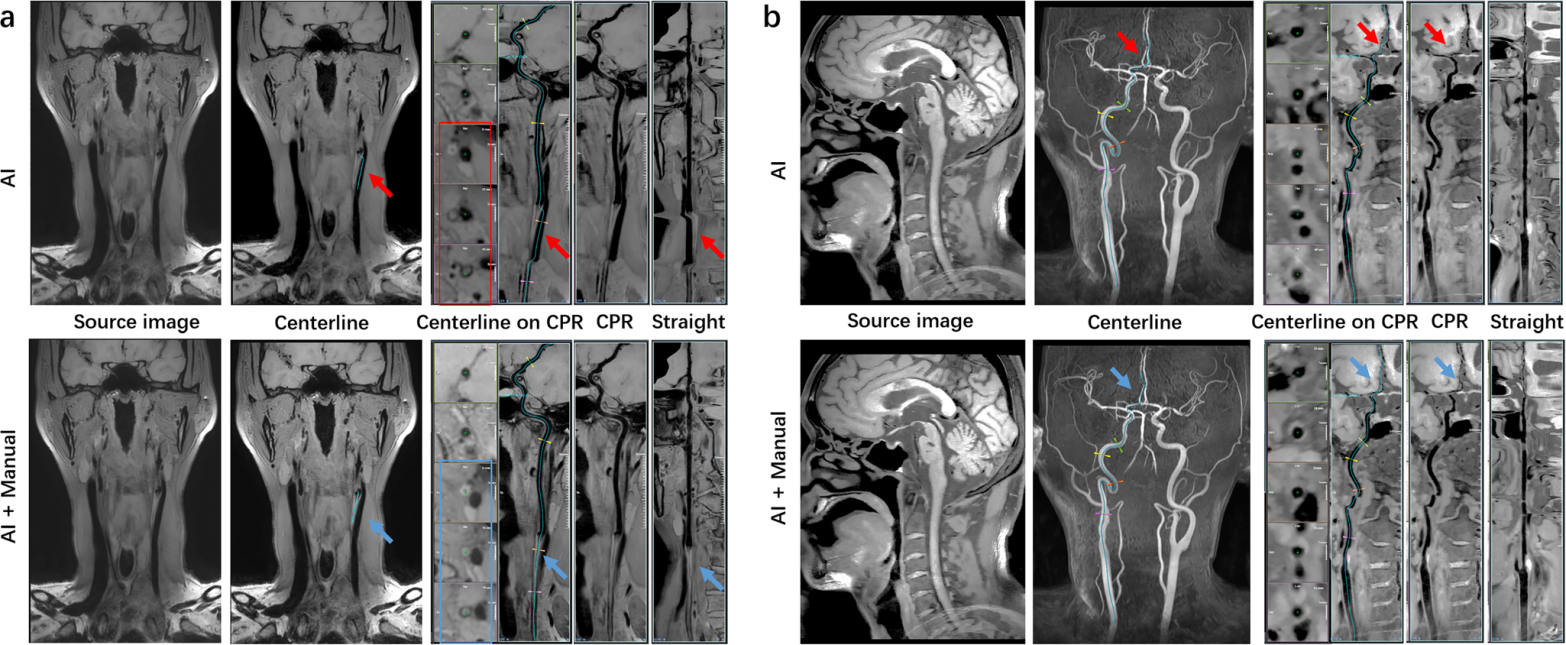
Erroneous postprocessing of VWI Assistant outputs and the corresponding corrections by manual modification based on VWI Assistant results. **a** A case of fibromuscular dysplasia. VWI Assistant misidentified the concomitant common carotid vein as the subtotal-occluded common carotid artery, resulting in an erroneous segmentation. **b** Incorrect recognition of the left anterior cerebral artery as the right anterior cerebral artery by VWI Assistant.

Figure 7a illustrates a case of incorrect vessel segmentation and recognition, where the VWI Assistant mistakenly identified the common carotid vein as the CCA in a patient with subtotal occlusion of the left CCA due to fibromuscular dysplasia. Figure 7b presents another case of incorrect vessel segmentation, where the VWI Assistant failed to accurately identify the left and right A2 segments of the ACA. In these instances, the manual process can be employed to adjust and correct the reconstruction based on the VWI Assistant’s results, improving accuracy while reducing post-processing time. Detailed steps for manual correction procedures are provided in a series of Supplementary Figures (Supplementary Fig. 1, Supplementary Fig. 2, Supplementary Fig. 3, Supplementary Fig. 4, Supplementary Fig. 5 and Supplementary Fig. 6).

### Comparing the performance of VWI Assistant with the manual process

**Fig. 8:**
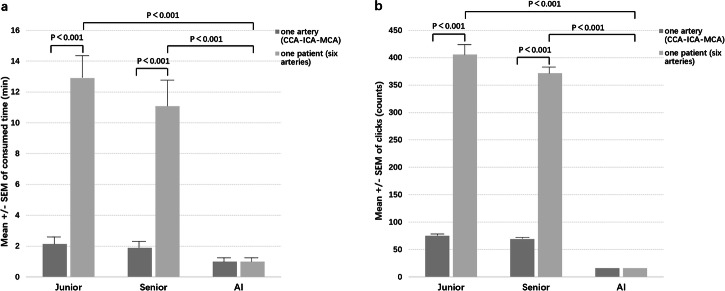
Comparison between VWI Assistant and pure manual processing in the clinical evaluation dataset. **a** Average time spent on the reconstruction of one artery in one patient by junior neuroradiologists, senior neuroradiologists, and VWI Assistant. **b** Average number of clicks during the reconstruction of one artery in one patient by junior neuroradiologists, senior neuroradiologists, and VWI Assistant.

In the clinical evaluation dataset, the average postprocessing time for one CCA-ICA-MCA using VWI Assistant was 0.99 ± 0.52 min, significantly less than the time required by a junior neuroradiologist (2.13 ± 0.77 min) and a senior neuroradiologist (1.89 ± 0.68 min). When reconstructing a series of cranio-cervical arteries from a single patient, the postprocessing time for VWI Assistant remained consistent at 0.99 ± 0.52 min, whereas the time for junior and senior neuroradiologists both substantially increased. The average time savings with VWI Assistant compared to the junior and senior neuroradiologists were 11.92 min and 10.09 min, respectively (*P* < 0.001, Fig. 8a). Additionally, the number of clicks required was drastically reduced, from 406.0 ± 26.6 by the junior neuroradiologist and 371.9 ± 16.3 by the senior neuroradiologist to just 16.2 ± 0.5 by VWI Assistant (*P* < 0.001, Fig. 8b).

We also assessed the inter- and intra-reader agreement for measuring the maximum stenosis rate and length of lesions in the ICA bifurcation, comparing measurements taken from VWI Assistant reconstruction, manual reconstruction, and only source image. In a randomly selected set of 34 arteries, all inter- and intra-reader agreement (ICC, 95% CI) for these measurements were excellent (all ICC > 0.900, *P* < 0.001), while the agreement was best for measurements taken after VWI Assistant postprocessing, as shown in Table 3.

**Table 3:** The inter- and intra-reader agreement of lesion stenosis and length (*n* = 34 arteries)

Variable	Inter-reader agreement			Intra-reader agreement		
	ICC	M ± SD	*P* value	ICC	M ± SD	*P* value
Maximum Stenosis Rate						
AI processing	0.988 (0.977, 0.994)	0.029 ± 0.028	<0.001	0.994 (0.988, 0.997)	0.018 ± 0.023	<0.001
Manual processing	0.968 (0.937, 0.984)	0.060 ± 0.038	<0.001	0.983 (0.967, 0.992)	0.046 ± 0.025	<0.001
Source images	0.938 (0.879, 0.968)	0.079 ± 0.052	<0.001	0.965 (0.931, 0.982)	0.061 ± 0.041	<0.001
Lesion Length						
AI processing	0.998 (0.997, 0.999)	0.982 ± 0.819	<0.001	0.999 (0.997, 0.999)	0.779 ± 0.901	<0.001
Manual processing	0.997 (0.994, 0.999)	1.327 ± 1.037	<0.001	0.998 (0.996, 0.999)	1.079 ± 0.972	<0.001
Source images	0.986 (0.972, 0.993)	2.803 ± 2.478	<0.001	0.988 (0.976, 0.994)	2.624 ± 2.047	<0.001

M ± SD: Mean difference ± standard error.

Numbers in parentheses are the 95% CIs.

The stenosis rate is expressed as a decimal.

The unit of length is millimeter (mm).

### Clinical application efficiency of VWI Assistant

**Fig. 9:**
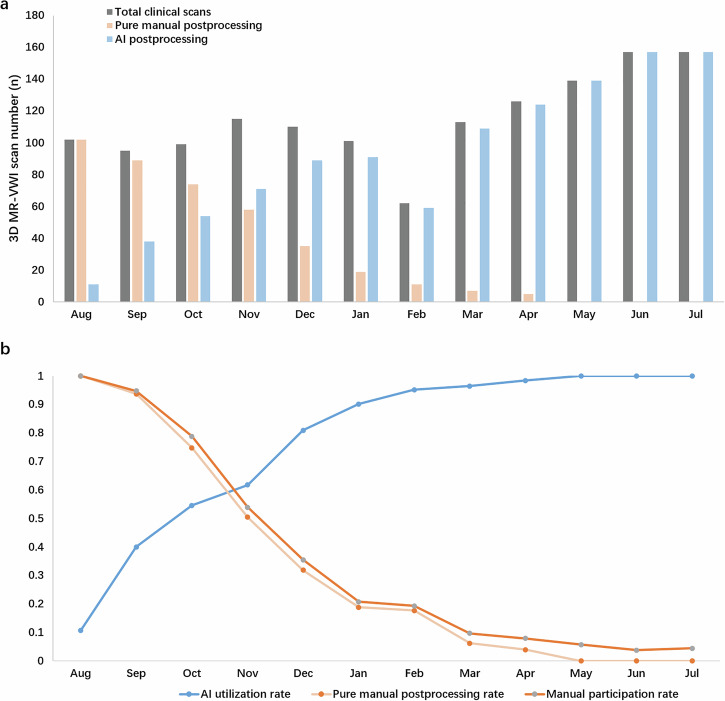
Clinical application of VWI Assistant from August 2023 to July 2024. **a** The number of clinical 3D MR-VWI scans, VWI Assistant postprocessing instances, and pure manual postprocessing instances. **b** The utilization rates of VWI Assistant, pure manual postprocessing rate, and manual participation rate.

The VWI Assistant was applied to patients who underwent 3D MR-VWI examinations at Renji Hospital and Hangzhou Bay Hospital from August 2023 to July 2024. The overall utilization rate of VWI Assistant was 79.9% (1099/1376), and the monthly utilization rate increased gradually, from 10.8% in August 2023 to 100.0% in July 2024, as shown in Fig. 9. Among them, 93.7% (1030/1099) were evaluated as qualified for clinical diagnostics by neuroradiologists.

## Discussion

In recent years, 3D MR-VWI has advanced from 2D imaging, offering unique advantages in large-scale lesion screening, stroke etiology clarification, and plaque risk assessment. As an important imaging tool for accurate stroke diagnosis and prevention, 3D MR-VWI has become indispensable. However, the intelligent algorithms for its postprocessing have lagged behind these technological advancements. This study aimed to develop and validate a deep learning algorithm, VWI Assistant, to enable efficient 3D MR-VWI postprocessing. We assessed its applicability across different disease scenarios and imaging manufacturers, comparing its performance with that of neuroradiologists. The neuroradiologists evaluated the reconstructed images, reporting a qualification rate of 92.9%. Importantly, VWI Assistant significantly reduced postprocessing time, decreasing the average time per patient over 10 min to just 0.99 ± 0.52 min. In this study, we successfully established a universal automatic imaging reconstruction system for 3D MR-VWI for different imaging protocols, equipment and populations based on large-sample multicenter data.

Previous studies have explored vessel segmentation and reconstruction techniques for 3D MR-VWI, though these efforts have been constrained by limited sample sizes. For instance, Wan et al. proposed an automated centerline extraction method utilizing TOF-MRA, which was subsequently co-registered with black-blood sequences to enable indirect 3D MR-VWI reconstruction via an optimized U-Net architecture^23^. This approach was validated on a dataset comprising MR vessel wall images from 47 healthy volunteers and 20 atherosclerotic patients. Similarly, Xu et al. introduced a convolutional neural network (CNN)-based algorithm for direct 3D reconstruction of vessel walls from black-blood sequences in the MATRIX study, employing a cohort of 124 patients with atherosclerotic plaques^24^. In another study, Shi et al. developed a CNN-driven automated segmentation framework for vessel wall analysis using 3 T Siemens MR imaging data from 56 participants^18^. In contrast, the present study significantly expands the dataset, enhances its diversity, and assesses the application value of the VWI Assistant in real clinical scenarios compared to previous efforts. The VWI Assistant model was trained on data from different black-blood techniques, such as VISTA, SPACE, MATRIX, and CUBE, obtained from leading MRI manufacturers. Furthermore, the dataset covered a wide range of clinical scenarios, including normal arteries, atherosclerotic stenosis, occlusion, and aneurysm, collected from multiple centers. In the clinical evaluation dataset, VWI Assistant demonstrated strong performance across these different conditions, with no significant difference in output quality compared to manual reconstruction.

In this study, we utilized multiple sequences for vessel segmentation and recognition, comparing the reconstruction outcomes across different sequence combinations. Although the combination of T1WI, TOF-MRA, and T1WI-CE required more processing time, it delivered the best performance in terms of algorithm accuracy and reconstruction quality. This outcome likely reflects the unique advantages of each sequence. TOF-MRA effectively delineates the alignment of the arterial lumen, particularly in the intracranial segments of the internal carotid artery^27^, which are often challenging to distinguish from surrounding structures in black-blood sequences. For large, occluded arteries, black-blood sequences offer insights into the extent, structure, and composition of the occlusion, which is important for preoperative planning^14,15,28,29^. Fresh thrombus appears as a high signal on T1WI sequences, enabling straightforward identification and tracing^30,31^. For occlusions without fresh thrombus, T1WI-CE sequences aid in visualizing the path of the occlusive segment either directly through enhancement of the occluded segment or indirectly through enhancement of the vessel wall of the occluded segment^15,32^. By integrating these diverse sequences, the model produced more accurate postprocessing images, enhancing its ability to handle complex reconstruction scenarios. In addition, the multi-sequence reconstruction retains the multi-sequence advantage of 3D MR-VWI, which is conducive to the further development of intelligent tools for lesion detection and analysis on this basis^10,33,34^.

Automatic reconstruction of occluded arteries on 3D MR-VWI remains a significant challenge, as few studies have developed deep learning models specifically for occlusions. In this study, in addition to employing the multiple sequences for reconstruction mentioned above, we incorporated a large dataset of occlusions into the model training to ensure accurate extraction and reconstruction of occluded vessels. Despite these efforts, some occlusions, such as those seen in moyamoya disease, proved challenging for both VWI Assistant and manual tracing, leading to unavoidable poor-quality reconstructions^35^. Nonetheless, VWI Assistant successfully reconstructed the occluded arteries in most cases. In the clinical evaluation dataset, the qualification rate of occlusion reconstruction for VWI Assistant was 80.3%, nearly identical to the performance of manual output (P > 0.05).

AI models are highly homogeneous in image postprocessing and are immune to human error. Given the same sequence, an AI model will consistently produce identical reconstructed image, regardless of who inputs the data or when it is processed. This ensures greater consistency in diagnostic outcomes. In our study, we evaluated the maximum stenosis rate and lesion length at the ICA bifurcation using raw data, manual processing, and VWI Assistant postprocessing. The measurements obtained from VWI Assistant exhibited the highest consistency, underscoring the advantages of AI in standardizing the diagnostic results of 3D MR-VWI. Additionally, AI models are fast, efficient and labor-saving. In the application dataset, 93.7% of cases were evaluated as qualified for clinical diagnosis, with an average processing time of just 0.99 ± 0.52 min per subject. Currently, VWI Assistant greatly reduces time costs while for a small number of cases where VWI Assistant reconstruction was not effective, we took to assist manual correction on the basis of AI reconstructions to ensuring accuracy. As the AI continues to learn and improve, this proportion of manual correction is expected to decrease further in the future.

Our study had several limitations. First, VWI Assistant demonstrates suboptimal performance in extracting small distal intracranial arteries (e.g., branches post M2 segment of the middle cerebral artery), as the model was primarily designed for large- and medium-sized arteries, manifesting as reduced segmentation accuracy, compromised centerline extraction quality, and frequent instances of centerline crossover between adjacent distal vascular branches. Second, VWI Assistant exhibited reduced accuracy in reconstructing complex vascular pathologies such as fibromuscular dysplasia or Moyamoya disease, where intricate vascular morphology was prone to centerline deviations, necessitating manual corrections. Third, despite an over 90% reduction in processing time, more than 5% of cases still required manual adjustments due to insufficient representation of rare anatomical variants or low-quality images in training data, compounded by potential subjectivity in gold standard annotations.

In summary, VWI Assistant represents a practical and efficient solution for 3D MR-VWI reconstruction. It offers a time-saving, objective, and efficient alternative to traditional manual methods, streamlining the reconstruction process, reducing costs, and improving overall workflow efficiency.

## Methods

### Data preparation

The dataset for this study comprised 2099 3D MR-VWI scans, randomly collected from four institutions in China between January 2018 and July 2024. These included 1441 scans from Ren Ji Hospital, affiliated with Shanghai Jiao Tong University School of Medicine; 528 scans from Shenzhen Institute of Advanced Technology; 86 scans from Ningbo Hangzhou Bay Hospital; and 44 scans from South Taihu Hospital. This multi-center study was approved by the respective institutional ethics committees of all participating centers (Ren Ji Hospital, affiliated with Shanghai Jiao Tong University School of Medicine; Shenzhen Institute of Advanced Technology; Ningbo Hangzhou Bay Hospital; and South Taihu Hospital) and conducted in accordance with the Declaration of Helsinki. Informed consent to participate in this study was obtained from all participants. All 3D MR-VWI scans were acquired in DICOM format using 3.0 T MR scanners from Philips, Siemens, GE, and United Imaging. Imaging protocols included 3D black-blood (T1WI for short) sequences [e.g., 3D volume isotropic turbo spin-echo acquisition (3D-Vista), 3D motion-sensitized driven equilibrium prepared rapid gradient echo (3D-Merge), 3D sampling perfection with application optimized contrast using different flip angle evolution (3D-Space), 3D fast spin echo with an extended echo train acquisition (3D-Cube), and 3D modulated flip angle technique in refocused imaging with extended echo train (3D-Matrix)] and 3D TOF-MRA (head, neck, or combined head and neck MRA). Some patients also underwent contrast-enhanced (CE) MRA and CE 3D black-blood imaging (T1WI-CE for short).

A total of 118 scans (5.7%) were excluded due to poor image quality. The remained 1981 scans were included in this study. Of these, 748 scans were included for modeling development, 134 for clinical evaluation, and 1099 for clinical application.

During the training, the black-blood sequence model was trained using 524 T1WI data and 206 T1WI-CE data, with validation performed on 104 T1WI and 42 T1WI-CE data. The bright-blood sequence model was trained on 510 TOF-MRA data and validated on 102 TOF-MRA data. For algorithm testing, the black-blood sequence model was evaluated using 60 T1WI and 60 T1WI-CE data, while the bright-blood sequence model was tested on 120 TOF-MRA data.

During the clinical evaluation stage, we used retrospective data from 134 patients to assess the clinical efficacy of the VWI Assistant system. Image processing outcomes were scored using a 4-point scale to determine their suitability for clinical requirements. The same images were reconstructed manually by a junior neuroradiologist ( ≤ 5 years of experience) and a senior neuroradiologist ( > 5 years of experience). A highly experienced neuroradiologist, blinded to the imaging sources, independently assessed the reconstruction quality using the 4-point scale.

The CPR images of the same source images were manually reconstructed by a junior neuroradiologist ( ≤ 5 years of experience) and a senior neuroradiologist ( > 5 years of experience), respectively. The CPR images of neck and head arteries were scored using a 4-point scale to determine their suitability for clinical requirements. A highly experienced neuroradiologist, blinded to the imaging sources, independently assessed the reconstruction quality using the 4-point scale. The scoring criteria were as follows: Score 1 indicates correct vessel segmentation and recognition with an accurate centerline, clear vascular delineation, no artifacts, and an image that was easy to diagnose. Score 2 reflects correct vessel segmentation and recognition with minor artifacts, where the centerline deviates from the target vessel by no more than 25%, and the artery was partially unclear but sufficient for diagnosis. Score 3 denotes correct vessel segmentation and recognition but with obvious artifacts, where the centerline deviates from the target vessel by no more than 50%, making the artery unclear and insufficient for diagnosis. Score 4 represents incorrect vessel segmentation and recognition with unidentified vessels, severe artifacts, and the centerline deviation exceeding 50%, resulting in a vessel that was indistinguishable and unsuitable for diagnosis.

Clinical use validation involved assessing the performance of VWI Assistant in real clinical scenarios by analyzing its application to actual clinical datasets, including evaluating post-processing operations performed by neuroradiologists and measuring time savings. We further recorded the utilization rates of VWI Assistant, pure manual postprocessing rate, and manual participation rate under the impact of increasingly popularization of VWI Assistant. Pure manual postprocessing rate referred to the rate of manual postprocessing without using VWI Assistant for vessel reconstruction. Manual participation rate referred to the rate of pure manual postprocessing plus manual correction for unqualified postprocessing by VWI Assistant.

### Clinical labeling

To ensure accuracy in data annotation, we implemented a cross-checking process. Initially, the 1728 original sequence datasets were delineated and annotated using a dedicated plaque analysis software (uWS PlaqueTool, United Imaging Healthcare) to segment various arteries, including the common carotid artery (CCA), internal carotid artery (ICA), external carotid artery (ECA), anterior cerebral artery (ACA), middle cerebral artery (MCA), vertebral artery (VA), basilar artery (BA), and posterior cerebral artery (PCA). Four neuroradiologists with over 5 years of experience manually performed the annotations, with each annotation cross-checked by other radiologists. An annotation was considered valid only when the annotating and reviewing radiologists reached consensus. In cases of disagreement, a highly experienced radiologist with more than 15 years of experience acted as an arbitrator to determine the final annotation. Each case required approximately 20 min for labeling. For centerline annotations, we obtained the coarse annotation results of the 3D Mask through lumen segmentation, slice interpolation, and smoothing processing. These annotations were then used for training the vascular segmentation model.

### Data preprocessing and increment

To address the challenge of limited annotated data, we employed data augmentation techniques to expand the training dataset, thereby enhancing sample diversity and improving the robustness of the vascular segmentation model. The augmentation operations included displacement, truncation, left-right reversal, and simulation of occluded vessels. These techniques resulted in a six-fold increase in dataset size. Displacement and truncation were applied to account for variations in FOV size and position across different sequences. Left-right reversal was used to account for anatomical left-right vascular differences. Simulating occluded vessels aimed to enhance the robustness of the segmentation model in scenarios involving arterial occlusions.

### Model development

**Fig. 10:**
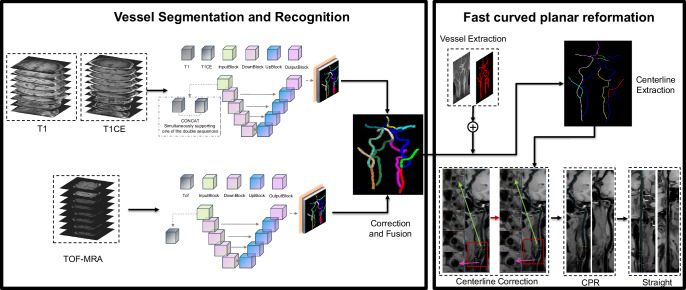
Rapid vessel segmentation and reconstruction framework of VWI Assistant. The framework of VWI Assistant comprises two primary computational phases: Phase I – Vessel segmentation and recognition and Phase II – Fast Curved Planar Reformation. For detailed information, please refer to the Methods section.

In this study, we developed an automated processing framework based on convolutional neural networks (CNN) for the segmentation, recognition, and rapid surface reconstruction of head and neck arteries. The framework consists of two main components, as shown in Fig. 10. The first component, vessel segmentation and recognition, utilizes enhanced multi-channel and single-channel CNNs to automatically segment both black-blood and bright-blood sequences. The segmentation results from each sequence model are then fused to generate the final segmentation, enabling accurate recognition of the vessel segments. In the second component, rapid vessel reconstruction is achieved by extracting principal arterial centerlines through skeletonization of the coarse segmentation results. Subsequently, the vessel extraction module is employed to obtain more comprehensive vascular segmentations in head-neck regions, compensating for potential deficiencies in the preceding vascular segmentation framework. Utilizing single-sequence (black-blood or bright-blood) segmentation outputs, the distance field -driven path tracing algorithm connects and extends the initially acquired main arterial centerlines to form complete vascular centerlines. Final refinement integrates lumen segmentation data across entire head-neck vascular segments, enabling precise correction of centerline trajectories in black-blood sequences.

Figure 10 demonstrates the ideal clinical setting under complete multi-sequence inputs. When imaging sequences are incomplete, the algorithm automatically identifies the available input sequences and executes post-processing reconstruction based on the existing sequence data. For example, when only bright-blood or black-blood sequences are available, the system automatically activates the corresponding dedicated processing module. For black-blood sequences with single T1WI or T1WI-CE inputs, dual-channel architecture enables robust reconstruction by duplicating available sequences across both processing channels.

### Training details

For black-blood sequence segmentation, a critical component of the overall framework, we employed an improved CNN architecture with Instance Normalization at the end of each 3D network to accelerate gradient convergence. The network includes an input processing unit, four down-sampling units, and four up-sampling units. The input processing unit uses a Conv3D layer to merge the dual input sequences, facilitating the integration of complementary information from both sequences. Each down-sampling unit employs BottResidual residual blocks to preserve input information, reducing information loss and enhancing information fusion. In the up-sampling units, residual blocks merge the down-sampling outputs at the same scale, ensuring that information from the same-scale is fully weighted, which accelerates the reduction of loss values. The output processing unit uses a 1 × 1 × 1 convolution to aggregate the feature map and obtain the final segmentation result.

The training was conducted on a machine equipped with 8 NVIDIA TITAN RTX GPUs (24GB memory), and the model experiments were implemented using the PyTorch framework. During training, the Adam optimizer was employed with a momentum of 0.9, an initial learning rate of 1e-3, exponential decay, and weight decay of 1e-5. The model converged and stabilized after 2500 epochs. The dice loss function was used to supervise the training, and segmentation performance was evaluated by comparing the probability of the segmentation output with the target gold standard’s dice coefficient.

During training, sequences that could not be strictly aligned after global rigid registration pose challenges, as the gold standard could only align one sequence during the merging of the two input sequences. To address this issue, we adopted a fixed alignment method for the first channel sequence, ensuring that the segmentation result was consistently aligned with the first channel, maintaining determinism in the results.

### Model effectiveness

The dice coefficient is used to evaluate the accuracy of the vessel coarse segmentation algorithm. This coefficient calculates the similarity between the segmentation result and the annotation result. The formula [Eq. (1)] for the dice coefficient is as follows:

1 $${\rm{Dice}}=\frac{2\left|X\cap \bar{X}\right|}{\left|X\right|+\left|\bar{X}\right|}$$

Here, $X$ and $\bar{X}$ represent the network predicted segmentation result and the manually annotated result, respectively.

The centerline accuracy is a custom measurement method that characterizes the accuracy of centerline extraction by calculating the ratio of accurately extracted centerlines to the overall centerline. The specific calculation formula [Eq. (2)] is as follows:

2 $${\rm{Centerline\; Accuracy}}=\frac{{\rm{length\; of\; extracted\; extractly\; centerline}}}{{\rm{length\; of\; groundtruth\; centerline}}}$$

The mean centerline distance (MCD) between the predicted vessel centerline and the annotated centerline is used to evaluate the accuracy of the prediction algorithm. The specific calculation formula [Eq. (3)] is as follows:

3 $${\rm{MCD}}=\frac{{\bf{1}}}{{\bf{2}}}\left(\frac{{\sum }_{{{\boldsymbol{v}}}_{{\boldsymbol{i}}}\in {{\boldsymbol{S}}}_{{\boldsymbol{G}}}}\mathop{{\mathbf{min }}}\nolimits_{{{\boldsymbol{v}}}_{{\boldsymbol{j}}}\in {{\boldsymbol{S}}}_{{\boldsymbol{P}}}}{\boldsymbol{d}}({{\boldsymbol{v}}}_{{\boldsymbol{i}}},\,{{\boldsymbol{v}}}_{{\boldsymbol{j}}})}{{{\boldsymbol{N}}}_{{\boldsymbol{G}}}}+\frac{{\sum }_{{{\boldsymbol{v}}}_{{\boldsymbol{j}}}\in {{\boldsymbol{S}}}_{{\boldsymbol{P}}}}\mathop{{\mathbf{min }}}\nolimits_{{{\boldsymbol{v}}}_{{\boldsymbol{i}}}\in {{\boldsymbol{S}}}_{{\boldsymbol{G}}}}{\boldsymbol{d}}({{\boldsymbol{v}}}_{{\boldsymbol{j}}},\,{{\boldsymbol{v}}}_{{\boldsymbol{i}}})}{{{\boldsymbol{N}}}_{{\boldsymbol{P}}}}\right)$$

Here, ${S}_{G}$ and ${S}_{P}$ represent the centerlines of the annotated vessel and the predicted vessel, respectively. The ${v}_{i}$ and ${v}_{j}$ represent the points on the annotated centerline and the predicted centerline, respectively. $d$ represents the distance between points on the centerline. ${N}_{G}$ and ${N}_{P}$ represent the number of points on the annotated centerline and predicted centerline, respectively.

### Statistical analysis

Categorical variables were expressed as frequencies (percentages, %) and continuous variables as the mean ± SD or as the interquartile range, depending on the data distribution. Student’s *t*-tests or Mann–Whitney *U*-tests were used for continuous data analysis, and Fisher’s test was applied to categorical data, as appropriate. Inter- and intra-reader agreement was assessed using the intraclass correlation coefficient (ICC). SPSS version 22.0 (IBM, Armonk) was used for data analysis. A *p* value of less than 0.05 was defined as significant, and all *p* values were two-sided.

## Supplementary information

Supplementary material

## Data Availability

Currently, the source imaging data from four centers cannot be made publicly accessible due to privacy protection but are available from the corresponding author [Huilin Zhao] on reasonable request.

## Code availability

The algorithm model developed in this study has been integrated into the post-processing software uWS Plaque Tool and uOmnispace MR Plaque Analysis, both by United Imaging Healthcare Co., Ltd. The VWI Assistant is accessible from the post-processing software uWS Plaque Tool and uOmnispace MR Plaque Analysis.
