# Supplementary material for: Rapid vessel segmentation and reconstruction of head and neck angiograms from MR vessel wall images
Source: NPJ Digit Med. 2025 Jul 28;8:483. doi: 10.1038/s41746-025-01866-x (PMC12304216; doi:10.1038/s41746-025-01866-x)
Supplement: Supplementary file 1 — Supplementary material [file 41746_2025_1866_MOESM1_ESM.pdf]

# Detailed steps for manual correction procedures

Figure 7a in the manuscript is a case of fibromuscular dysplasia. The left common carotid artery shows subtotal occlusion in the origin data. The VWI Assistant mistakenly identified the left common carotid vein as the left common carotid artery. We take the case of Figure 7a as an example and present the correction process with figures and text below：

**Supplementary Figure 1. Our AI-embedded software platform (uOmnispace MR Plaque Analysis).** Input the MR-VWI sequences. Automatically vessel segment, recognize and curved planar reformation.

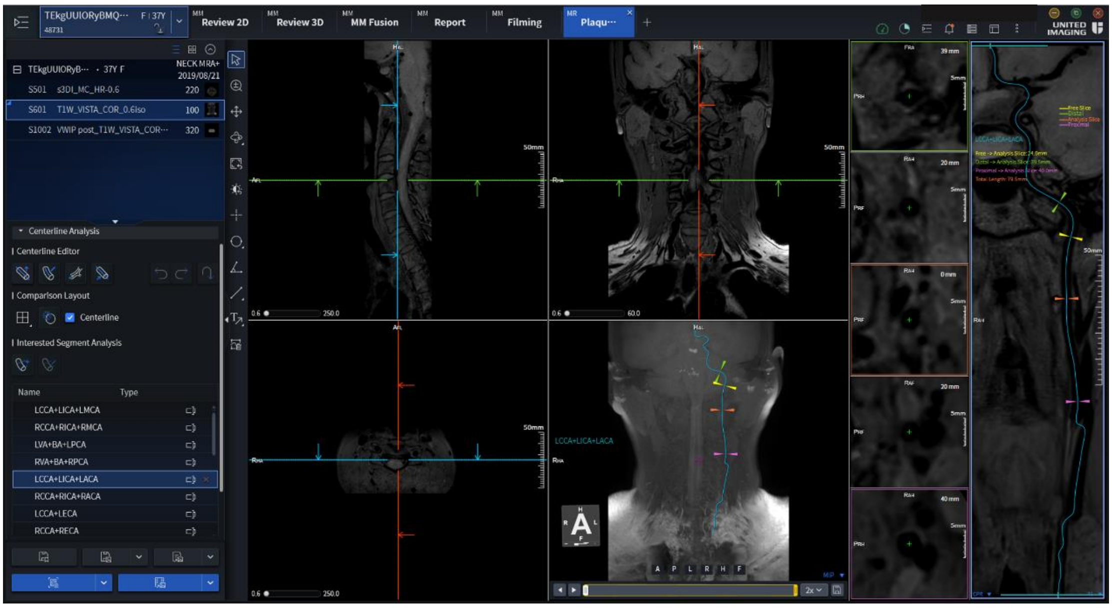


**Supplementary Figure 2. Manual review.** Find erroneous postprocessing. The red box shows the incorrect results of vessel segmentation and extraction. Click the "Centerline Editor" button indicated by the red arrow to perform manual correction.

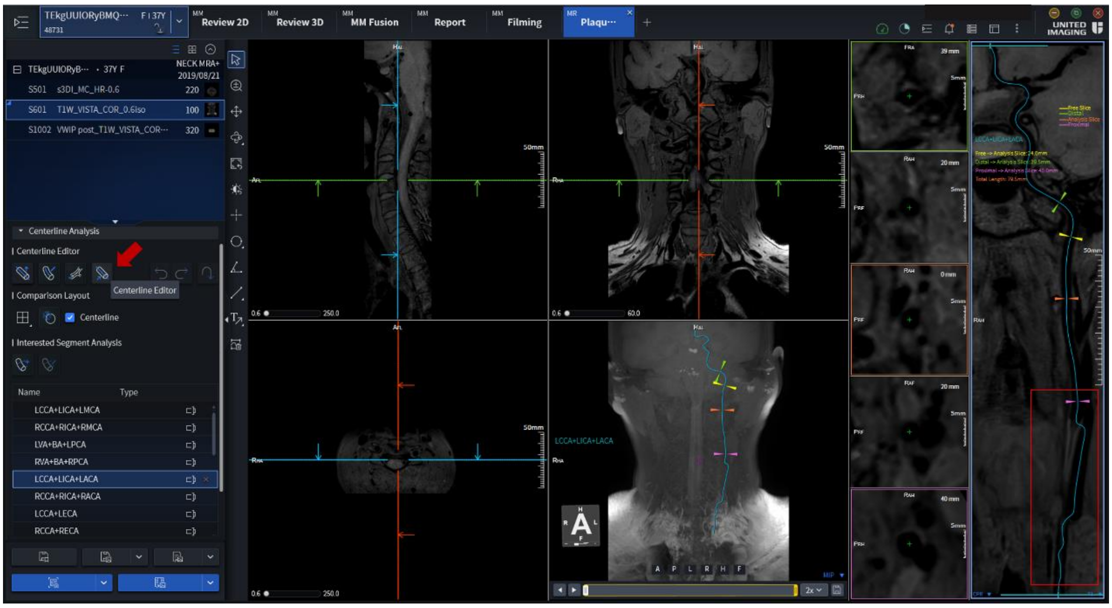


**Supplementary Figure 3. Manual correction processing.** Delete the incorrect part and keep the extracted correct part, as shown in the yellow box. Add angiogenesis point (indicated by the orange arrow) at the end of the correct part.

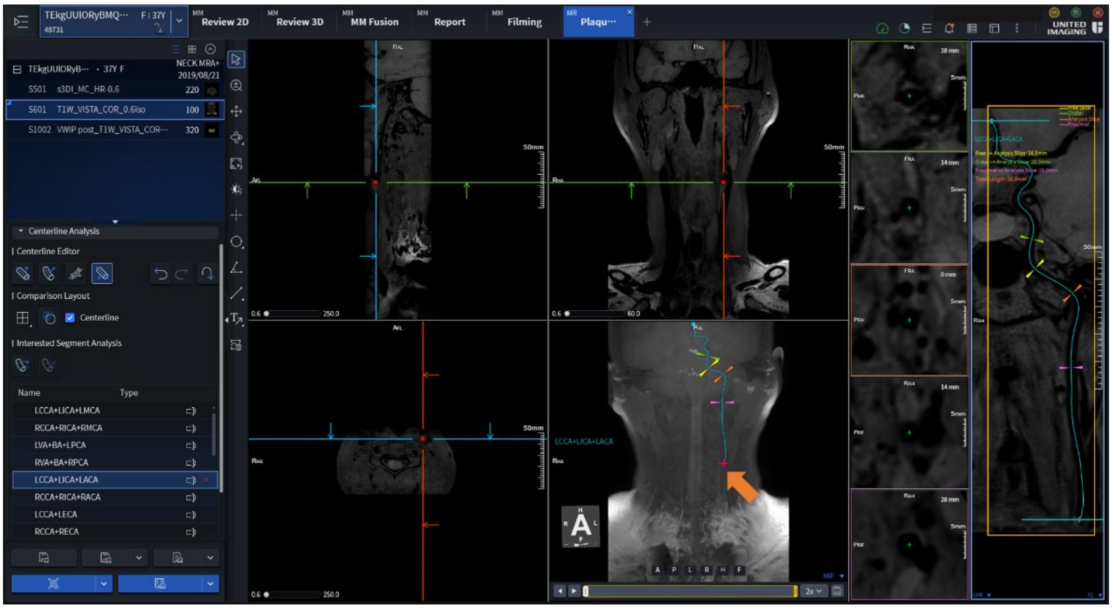


**Supplementary Figure 4. Track the target vessel.** Ensure the angiogenesis points (indicated by the orrange arrows) fall on the target vessel in the coronal, sagittal and transverse positions.

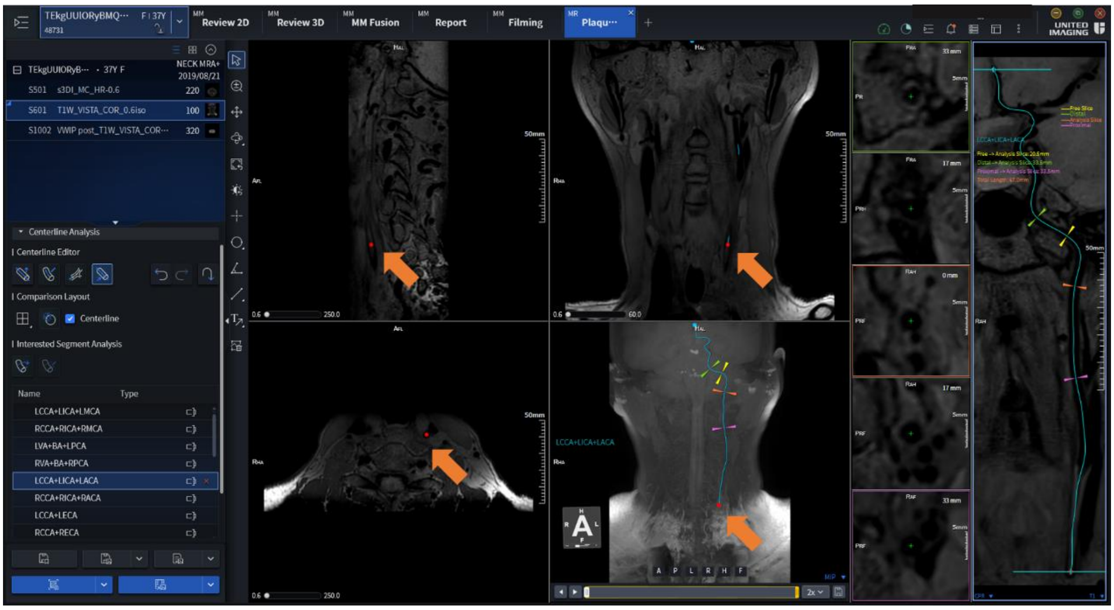

**Supplementary Figure 5. Generate correct centerline.** Click the "Centerline Editor" button again to generate a new and correct centerline of target vessel (green box).

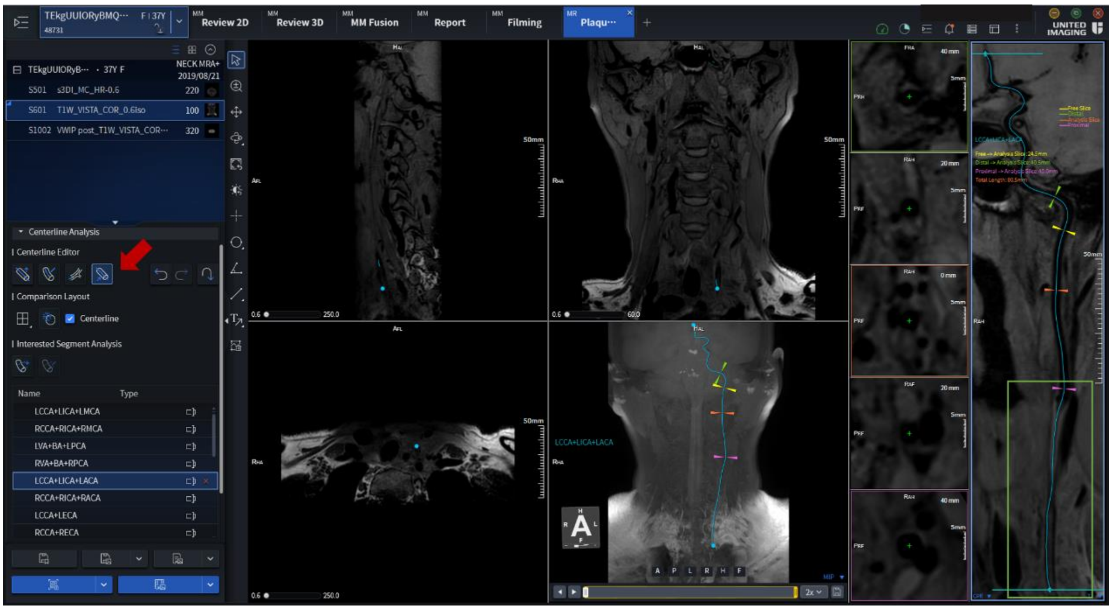

**Supplementary Figure 6. Manual correction completed.** Conceal the Centerline. Show the correctly reconstructed vessel after correction.

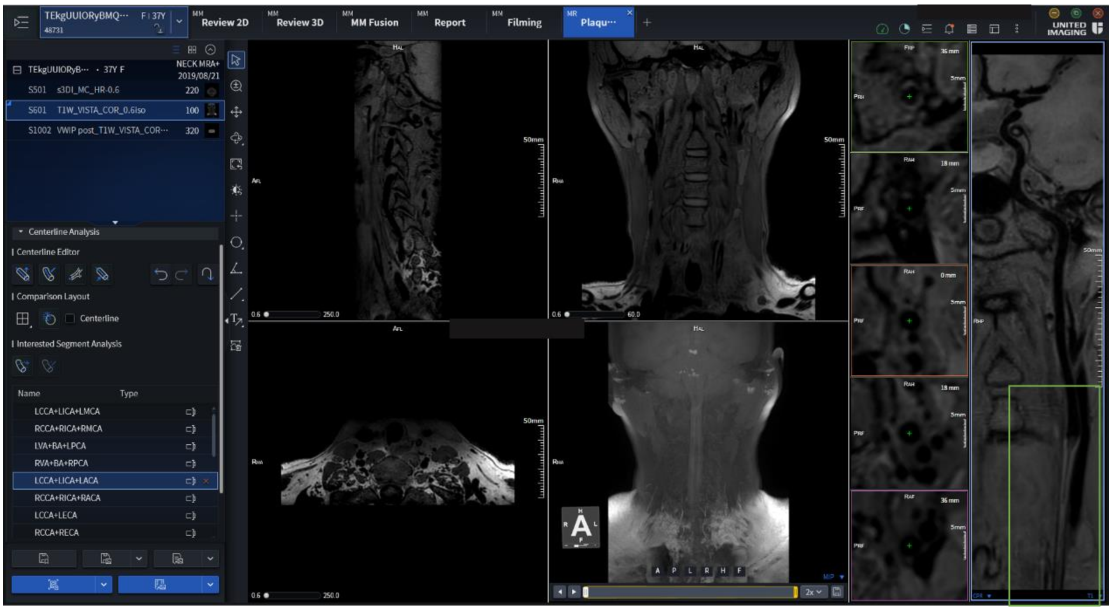


The above describes the whole manual correction process.
